# Rabbits as Animal Models for Anti-Tick Vaccine Development: A Global Scenario

**DOI:** 10.3390/pathogens12091117

**Published:** 2023-09-01

**Authors:** Arlex Rodríguez-Durán, Shafi Ullah, Luís Fernando Parizi, Abid Ali, Itabajara da Silva Vaz Junior

**Affiliations:** 1 Centro de Biotecnologia, Universidade Federal do Rio Grande de Sul, Avenida Bento Gonçalves, 9500, Porto Alegre 91501-970, RS, Brazil; arrodriguezdu@unal.edu.co (A.R.-D.); shafi_ullah@awkum.edu.pk (S.U.); luisfparizi@gmail.com (L.F.P.); 2 Programa de Pós-Graduação em Ciências Veterinária, Universidade Federal do Rio Grande de Sul, Avenida Bento Gonçalves, 9090, Porto Alegre 91540-000, RS, Brazil; 3 Grupo de Investigación Parasitología Veterinaria, Laboratorio de Parasitología Veterinaria, Universidad Nacional de Colombia, Carrera 45 No. 26-85, Bogotá 110911, Colombia; 4 Department of Zoology, Abdul Wali Khan University Mardan, Mardan 23200, Khyber Pakhtunkhwa, Pakistan; drabid@awkum.edu.pk; 5 Faculdade de Veterinária, Universidade Federal do Rio Grande de Sul, Avenida Bento Gonçalves, 9090, Porto Alegre 91540-000, RS, Brazil; 6 Instituto Nacional de Ciência e Tecnologia em Entomologia Molecular (INCT-EM), Rio de Janeiro 21941-853, RJ, Brazil

**Keywords:** antigen, humoral and adaptive response, immunization, rabbit, tick

## Abstract

Studies evaluating candidate tick-derived proteins as anti-tick vaccines in natural hosts have been limited due to high costs. To overcome this problem, animal models are used in immunization tests. The aim of this article was to review the use of rabbits as an experimental model for the evaluation of tick-derived proteins as vaccines. A total of 57 tick proteins were tested for their immunogenic potential using rabbits as models for vaccination. The most commonly used rabbit breeds were New Zealand (73.8%), Japanese white (19%), Californians (4.8%) and Flemish lop-eared (2.4%) rabbits. Anti-tick vaccines efficacy resulted in up to 99.9%. *Haemaphysalis longicornis* (17.9%) and *Ornithodoros moubata* (12.8%) were the most common tick models in vaccination trials. Experiments with rabbits have revealed that some proteins (CoAQP, OeAQP, OeAQP1, Bm86, GST-Hl, 64TRP, serpins and voraxin) can induce immune responses against various tick species. In addition, in some cases it was possible to determine that the vaccine efficacy in rabbits was similar to that of experiments performed on natural hosts (e.g., Bm86, IrFER2, RmFER2, serpins and serine protease inhibitor). In conclusion, results showed that prior to performing anti-tick vaccination trials using natural hosts, rabbits can be used as suitable experimental models for these studies.

## 1. Introduction

Ticks are obligate blood-sucking ectoparasites that parasitize a large number of terrestrial and semi-terrestrial vertebrates, including humans [1,2,3]. Although they have been considered cosmopolitan parasites, most tick species are restricted to specific habitats, especially in tropical and subtropical regions [4,5]. Ticks transmit a wide variety of pathogens, being the second most important vectors of pathogens affecting humans, and the main vector in domestic and wild animals [6,7].

Traditional methods to control these arthropods are mainly based on the use of synthetic acaricides [8,9,10]. However, the application of these products has disadvantages, including the selection of resistant tick populations, environmental contamination, and residues in products of animal origin such as milk and meat [11].

These issues raise the need to develop alternative control methods, including the selection of parasite-resistant breeds [12,13]; biological control using entomopathogenic fungi (*Metarhizium* spp., *Beauveria* spp.) [14,15]; entomopathogenic nematodes (*Heterorhabditidae* and *Steinernematidae*) [16,17]; regulator ants (*Solenopsis germinata*, *S. saevissima*, *Camponotus rengira*, and *Ectatomma quadridens*) [18,19]; pesticides [20,21]; and immunological control through the application of anti-tick vaccines [22,23,24]. 

Several proteins have been studied to date as candidates for the development of tick vaccines [25,26]. The immune response against target tick-derived proteins can affect the biological functions of ticks, such as feeding, blood digestion, protein regulation, water transport, reproduction, embryogenesis, immune response, and tick–pathogen interactions [27,28,29,30]. The first commercial anti-tick vaccine was obtained from the Bm86 protein [31]. The antigen hindered the feeding and reproductive ability of the *Rhipicephalus microplus* [31,32], and was used in two leading tick vaccines, TickGARD^®^ and GAVAC^®^ [33]. This landmark result, obtained by Willadsen et al. (1989) [31], paved the way for the investigation of new antigens and the development of vaccines that reduced infestations by *R. microplus* as well as other tick species.

The evaluation of tick vaccines in natural hosts has limitations, mainly due to the high costs of maintaining and using farm or wild animals in experiments. For this reason, animal models such as hamsters, guinea pigs, and rabbits are commonly used [34,35,36]. These animals have been used as models for basic and applied research, not only to test immune responses generated by anti-tick vaccines, but also to study resistance to chemical acaricides and tick-borne pathogen infection under laboratory conditions [37,38,39,40].

The use of hamsters, guinea pigs, and rabbits in tick vaccination experiments generally has low maintenance costs, minimal space requirements, short reproductive cycles and larger numbers of pups produced per year compared to some natural hosts [41,42,43]. However, there are distinct benefits and disadvantages to each of these models. For instance, the use of hamsters is limited by low blood volume, compared to the use of guinea pigs and rabbits [44,45]. On the other hand, guinea pigs have thick skin, which makes blood collection relatively difficult, sometimes even requiring anesthetic techniques to collect small volumes, in contrast to rabbits, which do not require anesthetic techniques for blood collection [46].

Another limitation in experimental animal models is the number of ticks that can be used when performing the infestation. Studies in rabbits have reported that these animals can support a higher burden of adult ticks [23,47] compared to mice, hamsters or guinea pigs [48,49]. Interestingly, the rabbit model was the first animal model used in several immunological studies and was crucial, for example, in the development of Louis Pasteur’s rabies vaccine in 1881 [50]. In 1976, the World Health Organization (WHO) [51] highlighted rabbits as among the most important laboratory animals for the study of different diseases [51,52,53,54]. The most common breeds of laboratory rabbits are derived from the European rabbit (*Oryctolagus cuniculus*) [55]. The American Rabbit Breeders Association (ARBA) enlisted 30 rabbit breeds that are used in experiments [56], among which the most used is the New Zealand white rabbit [30,42,57].

Laboratory rabbits have proven to be the most suitable and accessible hosts for all life-stages of various tick species during infestation and vaccination experiments [41,58]. This is because it has several advantages over the use of laboratory mice and rats, such as: (i) a longer life span than mice and rats [59]; (ii) a larger body size (up to four times larger than rats); (iii) higher blood volume, cell and tissue samples [60]; (iv) the production of copious antiserum [51,56]; and (v) easy maintenance and breeding [56].

Moreover, it is evident that rabbit-based experiments are more cost-effective when comparing trials conducted using large animals such as bovines. Various factors contribute to the overall costs, including animal prices, the extended maintenance period, a higher demand for feed, as well as the size and complexity of the animal facilities. Bovines require a greater amount of physical space and specialized infrastructure, along with large feed quantities. As a result, more demanding waste management systems are necessary for bovine experiments. Nonetheless, the determination of the real cost difference is difficult due to various local factors. This is because facilities, locations, and services vary among different regions and installations. Therefore, although rabbits incur lower costs, the precise amount varies.

Historically, the evaluation of tick-stimulated immune responses in rabbits began by studying the skin reactions caused by tick bites. A study by Trager observed that a single infestation of rabbits with *Dermacentor variabilis* larvae induced immunity that prevented subsequent larval infestations [61]. This work served as the basis for the subsequent use of rabbits as a model host for the development of anti-tick vaccines in the 1970s [62]. The main objective of this article was to review the current literature, underscoring the importance of laboratory rabbits as experimental models for the development of anti-tick vaccines, compare the immune responses developed in rabbits and the natural host, and evaluate the vaccine efficacy against potential anti-tick antigens.

## 2. Vaccination in Rabbits

**Figure 1:**
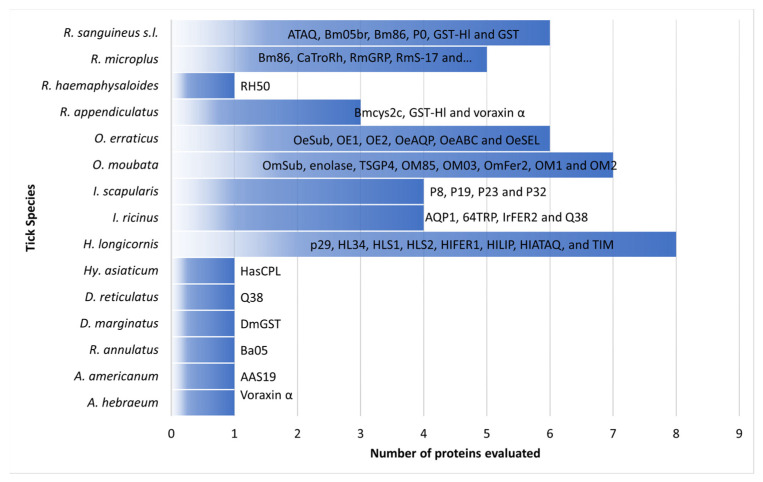
Tick-derived proteins evaluated in tick vaccination trials using rabbits as an animal model.

Rabbits are currently used as a model organism in anti-tick vaccines assays against ticks of the genera *Amblyomma*, *Dermacentor*, *Hyalomma*, *Haemaphysalis*, *Ixodes*, *Ornithodoros*, and *Rhipicephalus* (Figure 1) [23,35,63,64,65,66]. The following paragraphs will discuss the main trials carried out on rabbits for anti-tick vaccine development.

### 2.1. Haemaphysalis *spp.*

The tick *Haemaphysalis longicornis* tick is native to east Asia, with sparse distribution in Australia, New Zealand, and the U.S. [67,68]. It has a three-host life cycle, infesting cattle, and wild animals such as ungulates, lagomorphs, carnivores, and birds [69,70]. Immunological studies have shown different immunogenic proteins with the potential to develop a vaccine against *H. longicornis* from China and Japan. Japanese white rabbit and New Zealand breeds were mostly used in the infestation experiments.

**Figure 2:**
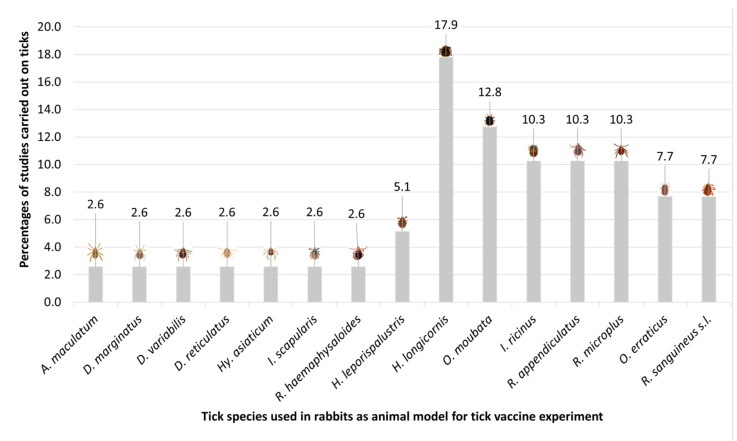
Different tick species and how often they are evaluated in studies using rabbits as models for vaccination experiments.

Eight proteins were evaluated for the purpose of vaccine development against *H. longicornis* using rabbits as an animal model (Figure 2). Wang et al. used New Zealand white rabbits to test the immune response against the lipocalin homologous protein of *H. longicornis*, obtaining a 60% reduction in the blood-feeding period of ticks, which would affect egg number, oviposition, and hatching rate [24]. In one experiment, Japanese white rabbits were immunized with protein 34 from *H. longicornis*, obtaining a partial reduction of this tick infestation [64,71].

### 2.2. Ornithodoros *spp.*

*Ornithodoros erraticus* and *Ornithodoros moubata* are nidicolous and endophilic argasid ticks that are widely distributed in different regions [72,73,74,75], and can intermittently feed on various vertebrates such as birds and canines [76,77]. Eight tick-derived proteins were evaluated for the development of vaccines against *O. erraticus* and *O. moubata* using rabbits as an animal model. Oleaga et al. tested the *O. moubata* ferritin 2 orthologues in New Zealand white rabbits, obtaining 71% efficacy for OmFer2, which corresponded to a decreased egg-hatching rate and in the subsequent number of emerging *O. moubata* larvae [78]. On the other hand, Pérez-Sánchez’s research group tested the immune response against aquaporin, showing moderate vaccine efficacy against *O. erraticus* [35].

**Figure 3:**
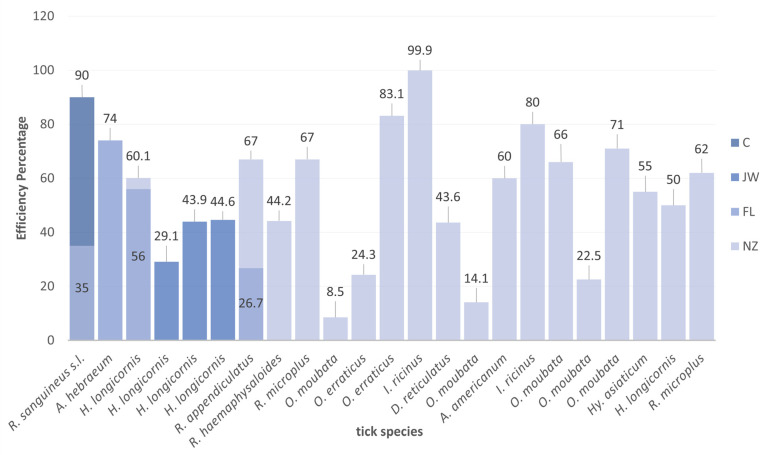
Vaccine efficiency (percent reduction of tick infestation) against different ticks, according to the breed of rabbits used for immunization. NZ: New Zealand. JW: Japanese white. C: Californians. FL: Flemish lop-eared rabbit.

A study carried out by Manzano-Román used New Zealand rabbits to test the protective effect induced by recombinant subolesin proteins against *O. erraticus* and *O. moubata*. The results showed a higher reduction in the *O. erraticus* oviposition when compared with *O. moubata* [79]. Manzano-Román et al. used the same rabbit breed to verify the vaccine efficiency of the synthetic peptides of subolesin/akirin, obtaining 83.1% vaccine efficacies against *O. erraticus* (Figure 3) [80]. These results show that rabbits presented anti-*O. erraticus* antibodies that recognized both subolesin proteins and protected against both argasid ticks with comparable efficacy.

### 2.3. Rhipicephalus *spp.*

*Rhipicephalus appendiculatus*, *Rhipicephalus microplus*, and *Rhipicephalus sanguineus* s.l. are medically important ixodid ticks of the genus *Rhipicephalus* [81]. *Rhipicephalus appendiculatus* is distributed in central, eastern, and southeastern Africa [82,83]. *Rhipicephalus microplus* and *R. sanguineus* s.l. are cosmopolitan ticks, distributed in the tropical and subtropical regions of the globe [6,84]. They present monoxene (*R. microplus*) and hetorexone (*R. sanguineus* s.l. and *R. appendiculatus*) lifecycles, preferring domestic hosts such as bovines, canines, and some wild animals, respectively. They feed on humans as incidental hosts [84,85].

New Zealand white, Californians, Japanese white, and Flemish lop-eared rabbit breeds were studied as model hosts for *R. appendiculatus*, *R. microplus*, and *R. sanguineus* s.l. in experiments carried out in Brazil, Cuba, Kenya, Japan, and Mexico, which proved to be successful in evaluating anti-tick proteins. Regarding the evaluation of proteins for the development of vaccines against *Rhipicephalus* species using rabbit as an animal model, a total of 15 molecules, to date, have been evaluated in rabbits for vaccine development against *Rhipicephalus* species, the most frequent tick genus in this kind of study.

The voraxin α homologue of the *R. appendiculatus* tick was used to immunize Japanese white rabbits, which resulted in a reduction in the weight of ticks, followed by a 50% reduction in egg mass [86]. On the other hand, a different study determined the vaccinal efficacy of rGST in New Zealand white rabbits, showing that rGST caused a reduction in the number of female *R. sanguineus* s.l. infestations [28].

Parizi et al. using the New Zealand white rabbit for immunization with the *R. microplus* cystatin 2c and reported a reduction in the number of fully engorged adult female ticks, causing damage to *R. appendiculatus* tissues such as the intestines, salivary glands and ovaries [23]. A study using Californian breed rabbits immunized with the P0 protein demonstrated a 90% reduction efficacy against *R. sanguineus* s.l.; a decrease in nymphs and larvae fed on vaccinated rabbits was observed [57]. In turn, Jittapalapong et al. determined the vaccine efficacy of recombinant *R. microplus* salivary gland serpins in New Zealand rabbits, obtaining an 83% reduction in adult *R. microplus* engorgement compared to the control [87]. These results indicate that this tick serpin is immunogenic for rabbits and suggest that this vaccine candidate antigen may confer protective immunity against the cattle ticks in this experimental model.

Additionally, for cattle ticks, Lagunes-Quintanilla et al. initially studied the recombinant peptide derived from the serpin RmS-17 protein as a vaccine in New Zealand rabbits in order to later vaccinate cattle. The results showed that the experimental vaccination reached 79% efficacy, limiting the number of infested adult ticks, the oviposition, and the fertility of the eggs [47]. Rabbits developed a strong humoral immune response expressed by high levels of anti-RmS-17 IgG. This was the first study evaluating the efficacy of the RmS-17 peptide against infestation by *R. microplus* ticks, and demonstrated in a rabbit model that it is both immunogenic and protective. A study used New Zealand rabbits to verify the vaccinal efficacy of the 50-kDa protein from *Rhipicephalus haemaphysaloides*, reporting a 74.7% protection in feeding ticks just 24 h after infestation [88]. The protein stimulated a specific protective immune response in tick-infested rabbits, demonstrating the success of rabbits as an animal model for these tick experiments.

### 2.4. Ixodes *spp.*

**Figure 4:**
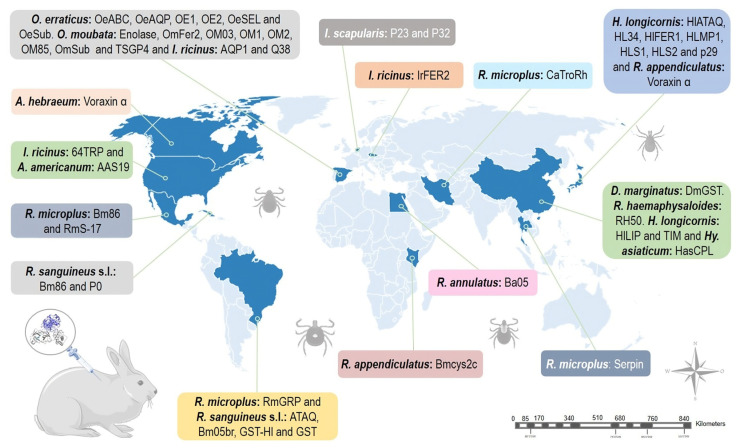
Geographical distribution of studies using rabbits as animal models to test anti-tick vaccines. Parts of the figures were drawn by using pictures from Servier Medical Art: http://smart.servier.com/ (accessed on 18 May 2023).

*Ixodes ricinus* and *Ixodes scapularis* are ixodid ticks that are characterized by a heteroxenous life cycle, and infest cattle, deer, dogs, and a wide variety of vertebrates, including humans [89,90,91]. The nymphal stage is most frequently responsible for transmitting pathogens to humans [92,93]. Of the 265 species of *Ixodes*, 55 are distributed in the neotropical regions of the planet [5]; however, *I. ricinus* and *I. scapularis* can be found only in the northern hemisphere [89]. Vaccination studies against *I. ricinus* and *I. scapularis* using the New Zealand rabbit breed were reported in the U.S., Spain, and the Netherlands (Figure 4) [48,94,95].

Five proteins were tested for the development of vaccines against *I. ricinus* and *I. scapularis*, using rabbits as an animal model. For instance, Contreras and de la Fuente evaluated the efficacy of CoAQP proteins against *I. ricinus* infesting New Zealand white rabbits and obtained an efficiency of 80% [95]. Meanwhile, Schuijt et al. evaluated P8, P19 and P23 proteins of *I. scapularis* in New Zealand white rabbits, demonstrating a reduction in feeding by infested nymphs in rabbits immunized with the cocktail antigens [94].

Contreras and de la Fuente used New Zealand rabbits as a vaccination model to describe the effect of the Q38 chimeric protein that conserved protective epitopes from *I. ricinus*. The vaccine had an efficacy of 99.9% in the reduction of *I. ricinus* larvae, with a cumulative effect in the reduction in tick survival and molting to the next life-stage [39]. Also, vaccination with *I. ricinus* recombinant protein ferritin 2 significantly reduced the number, weight, and fertility of ticks in vaccinated rabbits infested with *I. ricinus*, with an overall 98% vaccine efficacy [96]. These results demonstrate the feasibility of using ferritin 2 to develop vaccines to control tick infestations. A study showed that recombinant forms of the tick cement antigen *R. appendiculatus* 64P, act as a “double-acting” tick vaccine for *I. ricinus*, providing cross-protection for this ixodid tick, possibly by attacking antigens in the midgut and salivary glands of adults and nymphs. The tick mortality rate was 60%, and the results indicated the potential of 64TRPs as a broad-spectrum tick vaccine [48].

### 2.5. Dermacentor *spp.*

**Figure 5:**
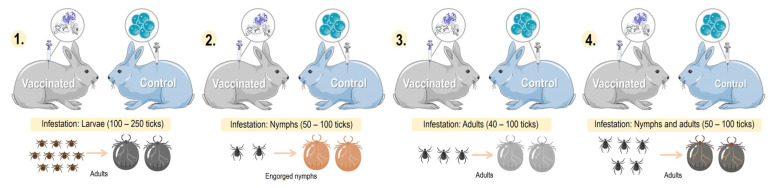
Comparison of different models of tick infestation in rabbits: 1. larval-stage tick infestation; 2. nymphal-stage tick infestation; 3. adult-stage tick infestation; and 4. nymphal- and adult-stage tick infestation. Parts of the figures were drawn by using pictures from Servier Medical Art: http://smart.servier.com/ (accessed on 21 February 2023).

*Dermacentor marginatus* is an ixodid tick that has a heteroxenous life cycle and a variety of hosts including canines, horses, and humans [97,98]. It is a tick with a cosmopolitan distribution, present mainly in the Nearctic, Palearctic, and Neotropic ecozones of the planet [99,100,101]. In the search for proteins for the development of a vaccine against *D. marginatus*, the New Zealand white rabbit was used as an animal model in infestations and vaccination experiments. A study infested New Zealand breed rabbits with *D. marginatus* after administering the last dose of the immunogen of GST, recording moderate vaccine efficacy against *D. marginatus* (Figure 5 and Table 1) [30].

## 3. Discussion

To date, 57 tick-derived proteins have been evaluated as potential anti-tick vaccines by studying the immunogenic responses generated using rabbits as an experimental model. Rabbit models for anti-tick vaccination trials have allowed for a better understanding of the physiological mechanisms of ticks infesting mammal hosts. For example, a study of the serpins HLS1, rHLS2, rSerpin, and RmS-17 in rabbits stimulated an immune response that affected the prolonged duration of feeding, increased mortality, and reduced oviposition in ticks like *H. longicornis* and *R. microplus* [47,64,87,102].

Globally, the use of rabbits has provided novel evidence on a vaccine based on salivary glycine-rich proteins in various medically important tick species. According to the findings obtained by Zhou et al., using rabbits immunized with the glycine-rich protein RH50, the protein was only expressed in the salivary glands of partially fed ticks and not in the salivary glands of unfed ticks or in the midgut, fat body, or ovary of partially fed ticks, in contrast to what was previously reported for p29 and Bm86 proteins [63,88,121].

Rabbits have been used as an immunization model to evaluate immunological responses to a given antigen (Q38, Bm86, GST, serpins and voraxin) against different tick species. For example, high vaccine efficacy against both *I. ricinus* and *D. reticulatus* was obtained with the chimeric protein Q38 containing subolesin/akirin [39].

Similarly, experiments on rabbits using voraxin α, a protein derived from the male tick and transferred to the female through copulation to stimulate female blood-feeding [104], have yielded vaccine efficiency by reducing feeding times in *Amblyomma hebraeum*. There is an amino acid sequence similarity between the voraxin α of *A. hebraeum* (85%) and that of *D. variabilis* (92%) and *R. appendiculatus* (85%) [86]. The immunization results could therefore potentially be similar, making this protein a good multispecies vaccine candidate. A reduction in the feeding time of ticks would also reduce salivation and, consequently, pathogen transmission, in addition to impairing oocyte development [104].

The use of rabbits as animal models in the discovery of anti-tick molecules has been fundamental in enabling the testing of these molecules before inoculation of the natural hosts. It was verified that rabbits present an immune response similar to that of the natural hosts. For example, the use of the ferritin 2 protein to immunize rabbits infested with *I. ricinus* (IrFER2) yielded an efficiency of 98%, while the efficiency of the same protein used in bovines infested with *R. microplus* and *R. annulatus* (RmFER2) was 64% and 72%, respectively [96]. Additionally, recombinant peptides derived from serpins showed efficacies against *R. microplus* of between 67% and 79% in rabbits [47,87]; in bovines, this protein showed an efficacy of 67% against *R. appendiculatus* [122].

Studies carried out with the Bm86 antigen in rabbits and cattle have shown that rabbits and cattle have a very similar reduction efficacy against *R. microplus*, obtaining a 62% reduction in rabbits [47], and a 60% reduction in cattle [96]. These results further indicate that rabbits are an excellent experimental model for initial vaccination experiments with anti-tick antigens, prior to the application of these in natural hosts such as bovines. Another benefit obtained by using rabbits as an animal model in research is the high recovery rate of fully engorged individuals of different tick species when carrying out infestations, compared to that of non-definitive domestic hosts. An experiment using *R. microplus* obtained a recovery of 33% in rabbits [123], compared to 3.7% in goats [124], 0.4% in dogs [125], and 1.8% in horses [126].

On the other hand, in a vaccination experiment on rabbits, Canales et al. reported for the first time that the recombinant bacterial membrane fraction containing the BM95-MSP1a chimera was effective in the control of *R. microplus* infestations. The BM95-MSP1a vaccine reduced oviposition and fertility of *R. microplus* similarly to that of the commercial vaccine Bm86, having a significantly greater immune response in vaccinated rabbits compared to the controls [106]. The results obtained in this experiment demonstrated that the rabbit is an excellent animal model with which to continue exploring new techniques or novel anti-tick antigens.

The infestation time of the *R. microplus* nymphal-to-adult stages on rabbits takes an average of 30 days to complete [127], while the time taken to complete these two life stages in bovines is between 14 and 20 days, on average [128]. These data indicate that the infestations of some tick species, such as *R. microplus*, in rabbits could be a valuable alternative animal model for the evaluation of candidate vaccines and new molecules with acaricidal activity against this ectoparasite. A possible explanation of why the life cycle takes longer in the nymphal-to-adult life stages of *R. microplus* in rabbits could be due to the inflammatory cellular response caused by tick bites at the beginning of feeding, which prevents them from accessing the blood source, which can in turn lead to increased mortality and feeding times [129]. Another aspect could be the strong competition between ticks due to the small physical body space for feeding provided by rabbits as a feeding model, which can cause the death of some ticks who are not able to adhere to the skin at the beginning of the infestation [127,130].

Rabbits immunized with 64TRP and infested with *R. sanguineus* s.l. or *I. ricinus* developed local inflammatory immune responses involving leukocytes, basophils, eosinophils, lymphocytes, mast cells, and macrophages. In turn, bovines immunized with 64TRP and challenged by *R. appendiculatus* showed dermal migration of dendritic cells, actively degranulating mast cells, basophils, and eosinophils [48]. These authors also found that the GST protein generated very similar inflammatory responses (mainly caused by eosinophils or mast cells) in hamsters, rabbits, and bovines [48]. Furthermore, infestation-only studies revealed a similar immune response against *R. appendiculatus*, with infiltration of neutrophils, macrophages, eosinophils, and basophils in both rabbits and cattle [54].

The immune responses generated by the different proteins studied in rabbits could vary depending on the challenges of ticks in immature or mature life stages. For example, the response generated by the p29 and HL34 proteins in the life stages of larvae, nymphs, and adults of *H. longicornis* fed on immunized rabbits suggests that these proteins may be involved in mediating key physiological functions in the tick [63,71]. Although mature and immature ticks commonly express native p29, their sensitivities to rabbit immune responses against rp29 appear to be different [63], while the native HL34 is expressed in both immature (larvae and nymphs) and adult ticks. It is thus likely that immunity against rHL34 is directed towards immature and mature ticks [71].

This result can be supported by Kemp et al. who recorded that *R. microplus* in immature and mature states have different sensitivities for acquiring resistance against anti-tick molecules. While there was severe intestinal damage in adult females and males feeding on cattle infested with *R. microplus*-derived antigens, there was no effect on tick larvae feeding on the same protected cattle [119]. Therefore, we can suggest that the different vaccine effects between immature and mature *H. longicornis* ticks fed on rp29-vaccinated rabbits could be consistent as well on natural hosts.

Additionally, studies on rabbits have allowed us to broaden our knowledge about the “exposed” and “hidden” antigens of anti-tick proteins. For example, it was reported that HLS1 acts on the expression of hidden antigens, inhibiting the secretion of rHLS1 in rabbits during feeding [64]. Also, 64TRP isoforms were characterized as “dual-acting” anti-tick proteins against *R. sanguineus* s.l. and *I. ricinus*; they target both “exposed” and “hidden” antigens, preventing attachment, and feeding by affecting the feeding site, as well as cross-reacting with ‘hidden’ midgut antigens, resulting in the death of engorged ticks [48].

Only a few studies focused on identifying molecules that affect male feeding or reproduction. One of the proteins that was identified and tested in rabbits is voraxin [105]. The preliminary vaccination of rabbits with voraxin α demonstrated humoral immunity and conferred protective immunity against female *R. appendiculatus* ticks, resulting in reduced feeding weights [86]. This may indicate that the antibodies against voraxin α affect female ticks of the same species. This same result was observed in the female *A. hebraeum*, which was studied in infestations on rabbits immunized with voraxin α, where a reduction in feeding of up to 72% was obtained compared to that of the engorged ticks on the control rabbits [104]. These results suggest that rabbits may be a good model not only for the study of anti-tick molecules that act on females, but also for the molecules that act on males.

Results obtained from the study of the tick saliva proteome have shown a variety of proteins that protect ticks against host immune responses and antihemostatic mechanisms [131,132,133,134,135,136]. This is because, during hematophagy, tick salivary glands undergo remarkable growth and differentiation, accompanied by a significant increase in the synthesis of different proteins [137]. Tirloni et al. identified 187 tick and 68 bovine proteins in the saliva proteome of *R. microplus*, demonstrating that *R. microplus* saliva is rich in hemolipoproteins, lipocalins, peptidase inhibitors, antimicrobial peptides, glycine, and maintenance proteins [133]. These proteins, together with pharmacological bioactive lipids, can counteract the host’s defenses and hemostatic mechanisms [131,138], while the host physiological systems can trigger changes in the feeding activity of ticks [139] by stimulating proteins to limit host defense mechanisms [140].

Another study by Tirloni et al. examined the saliva proteome of non-fed adult ticks of *I. scapularis* and *A. americanum* stimulated in different hosts, including rabbits, dogs and humans, identifying a total of 276 proteins in *I. scapularis* and 340 proteins in *A. americanum*. Among these proteins, 55 (*I. scapularis*) and 67 (*A. americanum*) belonged to the same functional classes [141]. These data suggest that *A. americanum* and *I. scapularis* use a core set of functionally similar proteins that regulate key host defense pathways to successfully feed. *I. scapularis* saliva had a high abundance of proteins related to heme/iron metabolism, followed by extracellular matrix/cell adhesion, oxidative metabolism/detoxification, cytoskeletal metabolism, proteasome machinery, nuclear regulation, conserved protein with unknown function, modification proteins, protein synthesis machinery proteins, and transport/storage. In turn, *A. americanum* saliva had a high abundance of extracellular matrix/cell adhesion proteins and proteinase inhibitors, followed by immune-related heme/iron metabolism, energy protein metabolism, cytoskeletal, protein synthesis machinery and proteasome machinery [141].

The above results indicate that these two tick species could inject the same protein at different levels into different hosts, and that the protein composition of the saliva of different tick species feeding on the same host is likely to be different. Furthermore, the results suggest that ticks of the same species differentially express tick salivary proteins when stimulated to start feeding on different hosts, expressing unique protein profiles in their saliva. There is evidence that ticks differentially express specific sets of genes when stimulated to start feeding [142,143]. For example, *A. americanum* saliva proteins contain a diversity of protease inhibitors (PI), expressing a total of 155 PI proteins belonging to eight families. Approximately 74% of these PI (115/155) were secreted into saliva within the first 120 h of feeding, indicating that the functions of the PIs are associated with the regulation of the early stages of feeding in *A. americanum*, which could also include the transmission of TBD agents by *A. americanum* [144].

On the other hand, Tirloni et al. identified differences in the expression of proteins in the development stages of nymphs and adult females of *H. longicornis*, obtaining 30 proteins in the saliva of nymphs, 74 proteins in the saliva of fully engorged adult females, and 31 proteins that were detected at both stages [134]. Proteins expressed in adult saliva may be related to exposure to different vertebrate hosts and the different stages of development, leading to changes in the dynamics of salivary transcription [132,145]. Taken together, those studies demonstrate that, even though the protein profile of tick saliva is strongly influenced by the host they infest, rabbits can be used as an alternative non-natural host to continue exploring and describing proteins that serve as candidates for tick vaccines.

## 4. Conclusions

The current review summarized the evaluation of 57 antigens as anti-tick vaccines in different rabbit breeds. These breeds include New Zealand, Japanese white, Flemish lop-eared, and California rabbits. The most widely used is the New Zealand breed, in countries located in Africa, Asia, America, Oceania, and Europe. Rabbits are not the natural host of most tick species; however, this has not been a limitation in obtaining vaccination results very similar to those of natural hosts for different tick species. Likewise, the use of rabbits has provided valuable insights on the immunological responses generated by novel antigens prior to vaccination trials in natural hosts. Rabbits stand out among other animal models used in vaccination experiments because they are suitable and commercially accessible alternative hosts for challenging the larval, nymphal, and adult life-stages of various tick species.

## Figures and Tables

**Table 1:** Use of rabbits as animal models for anti-tick vaccine experiments.

No	Experiment/Molecule Name	# of Rabbits	Rabbitsbreed	Tick Species	Tick Stages	Immunization	Tick per Rabbit	% Reduction	Reference
1	*Haemaphysalis longicornis* lipocalin (HlLIP)	6	NZ	*H. longicornis*	Adults	3	46	60.1	[24]
2	Glutathione S-transferase GST-cocktail	6	NZ	*R. sanguineus* s.l.	Adults	3	60	35	[28]
3	*Dermacentor marginatus* S-transferase (DmGST)	6	NZ	*D. marginatus*	Nymphs and adults	3	110	43.6	[30]
4	Aquaporin of *Ornithodoros erraticus* (OeAQP) and selenoprotein T of *Ornithodoros moubata* (OeSEL)	9	NZ	*O. erraticus* and *O. moubata*	Nymphs and adults	3	180	47.5 and 22.5	[35]
5	Q38	3	NZ	*I. ricinus* and *D. reticulatus*	Larvae	2	200	99.9 and 43.6	[39]
6	RmS-17 and Bm86	6	NZ	*R. microplus*	Adults	3	120	79 and 62	[47]
7	64TRP	14	NR	*I. ricinus*	Adults	3	30	NA	[48]
8	Evaluation of the immune response	NR	NZ	*H. leporispalustris*	Nymphs	1	NR	NA	[52]
9	Whole tick tissues collected from *Amblyomma maculatum*	8	NZ	*A. maculatum*	Nymphs and adults	2	75	NA	[53]
10	Attachment sites of *Rhipicephalus appendiculatus*	5	NZ	*R. appendiculatus*	Adults	3	80	NA	[54]
11	P0 protein and Bm86	10	C	*R. sanguineus* s.l.	Nymphs and adults	4	400	90	[57]
12	Evaluation of the immune response	6	NR	*D. variabilis* and *H. leporispalustris*	Larvae	1	159	NA	[61]
13	Native protein (p29)	10	JW	*H. longicornis*	Nymphs and adults	3	2110	56	[63]
14	*Haemaphysalis longicornis* serpin 1 (HLS1)	4	JW	*H. longicornis*	Nymphs and adults	2	120	43.9	[64]
15	*Haemaphysalis longicornis* ferretin 1 (HlFER1)	3	JW	*H. longicornis*	Adults	1	50	NA	[65]
16	Triosephosphate isomerase homologue from *Haemaphysalis longicornis* (HlTIM)	27	NZ	*H. longicornis*	Adults	1	92	50	[66]
17	*Haemaphysalis longicornis* protein 34 (HL34)	4	JW	*H. longicornis*	Nymphs and adults	2	115	29.1	[71]
18	Ferritin 2 in *Ornithodoros moubata* (OmFer2)	6	NZ	*O. moubata*	Nymphs and adults	3	95	71	[78]
19	Subolesin *Ornithodoros erraticus* and *Ornithodoros moubata* (rOeSub and rOmSub)	9	NZ	*O. erraticus* and *O. moubata*	Nymphs and adults	3	90	8.5 and 24.3	[79]
20	Subolesin/akirin orthologues of *Ornithodoros erraticus* (OE1, OE2 and OM1)	3	NZ	*O. erraticus*	Adults and nymphs	3	200	48.6, 83.1 and 50.3	[80]
21	rVoraxin from *Rhipicephalus appendiculatus*	3	JW	*R. appendiculatus*	Adults	3	60	26.7	[86]
22	Serpin	6	NZ	*R. microplus*	Larvae	3	500	67	[87]
23	RH50	6	NZ	*R. haemaphysaloides*	Nymphs and adults	3	120	74.7	[88]
24	Salivary antigens P8, P19, P23 and P32	3	NZ	*I. scapularis*	Nymphs	3	50	NA	[94]
25	CoAQP	6	NZ	*I. ricinus*	Larvae	2	200	32 and 80	[95]
26	IrFER2	4	NR	*I. ricinus*	Nymphs	3	50	98	[96]
27	*Haemaphysalis longicornis* serpin-2 (HLS2)	4	JW	*H. longicornis*	Nymphs and adults	2	160	44.6	[102]
28	Tick egg yolk protein (vitellin)	4	JW	*O. moubata*	Nymphs and adults	4	112	NA	[103]
29	Voraxin of *Amblyomma hebraeum*	2	FL	*A. hebraeum*	Adults	3	62	74	[104]
30	Protein 05 from *Boophilus annulatus* (Ba05)	1	NR	*B. annulatus*	Larvae	NA	NA	NA	[105]
31	Recombinant BM95-MSP1a fusion protein and Bm86	16	NZ	*R. microplus*	Adults	1	50	65.5 and 55.9	[106]
32	REnolase	3	NZ	*O. moubata*	Adults	3	90	NA	[107]
33	*Ornithodoros moubata* salivary lipocalin (TSGP4)	6	NZ	*O. moubata*	Adults and nymphs	3	100	14.1	[108]
34	*Amblyomma americanum* serine protease inhibitor 19 (AAS19)	2	NZ	*Amblyomma americanum*	Adults	2	40	60	[109]
35	ATAQ protein from *Rhipicephalus microplus*	9	NZ	*R. sanguineus* s.l.	Adults	3	NR	47	[110]
36	*Rhipicephalus microplus* ticks from Brazil (Bm05br)	1	NZ	*R. sanguineus* s.l.	Adults	3	NR	NA	[111]
37	Glutathione S-transferase from *Haemaphysalis longicornis* (GST-Hl)	14	NZ	*R. sanguineus* s.l. and *R. appendiculatus*	Nymphs and adults	3	190	67	[112]
38	OM85 and OM03	6	NZ	*O. moubata*	Nymphs and adults	3	40	20.7 and 66.1	[113]
39	Cathepsin L and tropomyosin proteins derived from *Rhipicephalus microplus* (CaTroRh)	6	NZ	*R. microplus*	NA	3	NA	NA	[114]
40	Cathepsin L from *Hyalomma asiaticum* (HasCPL)	6	NZ	*Hy. asiaticum*	Larvae	3	250	55	[115]
41	ATAQ in *Haemaphysalis longicornis* (HlATAQ)	2	JW	*H. longicornis*	Adults	2	30	NA	[116]
42	Hexokinase of *Haemaphysalis longicornis* (HlHK)	12	NZ	*H. longicornis*	Adults	3	46	65.6	[117]
43	Acid tail salivary protein (OeATSP), multiple coagulation factor deficiency protein 2 homolog (OeMCFD2), Cu/Zn-superoxide dismutase (OeSOD) and sulfotransferase (OeSULT) of *Ornithodoros erraticus*	6	NZ	*O. erraticus*	Nymphs and adults	3	95	58.3	[118]
44	RmGRP	N/A	NZ	*R. microplus*	N/A	9	N/A	NA	[119]
45	*Haemaphysalis longicornis* metalloprotease (HLMP1)	3	NR	*H. longicornis*	Nymphs and adults	3	120	15.6 and 14.6	[120]

NR: Not reported. NA: Not applicable. NZ: New Zealand. JW: Japanese white. C: Californians. FL: Flemish lop-eared rabbit, and No: Number of ticks per rabbit.

## Data Availability

Not applicable.
